# The Role of Hedgehog Pathway in Uterine Leiomyosarcoma

**Published:** 2021-08-23

**Authors:** Natalia Garcia, Mara Ulin, Ayman Al-Hendy, Qiwei Yang

**Affiliations:** 1 Greehey Children’s Cancer Research Institute, UT Health San Antonio, San Antonio, Texas, United States; 2 Department of Obstetrics and Gynecology, Sinai Health System at Chicago Illinois, Illinois, United States; 3 Department of Obstetrics and Gynecology, University of Chicago, Illinois, United States

## DESCRIPTION

The uterine Leiomyosarcoma (LMS) represents 3%-7% of all uterine cancers. This rare tumor has an annual incidence of 0.8 per 100,00 women. Unfortunately, LMS is well known for its poor prognosis due to its high recurrence rate and resistance to the currently available treatment options. These features open the field for new therapeutic options.

The Hedgehog (HH) pathway is a mechanism and a signaling cascade that directs the development of embryonic cells in animals. Since its first discovery in the 1980’s, the HH pathway has been identified to play a crucial role in many biological processes, including embryonic development, tissue differentiation, and tissue maintenance. The activation of the HH pathway promotes GLI translocation into the nucleus leading to the overactivation of several target genes, which regulate cell differentiation, proliferation and apoptosis, cell cycle, DNA damage, angiogenesis, and adhesion, contributing to the pathogenesis of diseases including cancer.

The activation of the HH signaling pathway has been characterized in several types of female cancer, including breast, ovarian, endometrium, cervical, and uterine leiomyosarcoma [1]. Several compounds have been identified to inhibit the HH signaling pathway and can be categorized as HH ligand inhibitors (HH neutralizing antibodies and small molecule Robotnikinin), SMO antagonists {(cyclopamine and its derivatives (IPI-926 and Cyc-T)} and synthetic compounds such as Vismodegib and Sonidegib), and GLI transcriptional inhibitors (Gant58 and Gant61). Clinical applications of HH pathway inhibitors have shown to significantly benefit preclinical and clinical studies to treat several types of female cancer.

The role of the HH pathway in uterine LMS was first described in 2016, where we characterized the protein expression of HH components in LMS compared to benign uterine fibroids and myometrium. We detected elevated expression of SMO and GLI1 in LMS compared to uterine fibroids and normal myometrium. We also observed that HH and protein levels of SUFU, the negative regulator of HH pathway, were correlated with poor prognosis in leiomyosarcoma [2]. However, we did not observe the differential protein expression of SUFU among myometrium, uterine fibroids, and leiomyosarcoma cells.

Targeting SMO and the GLIs have been demonstrated to be a valuable strategy to block the HH signaling pathway activity and suppress the tumor progression. In addition, SMO and GLI inhibitors have been shown to exert an anti-cancer activity both *in vitro* and *in vivo* on different types of cancer. Several SMO inhibitors are approved by the FDA and have been tested in clinical trials showing promising results.

To further determine the role of the HH pathway in LMS, we evaluated the effects of pharmacological inhibition of SMO (LDE225 and GDC0449) and GLI (Gant58 and Gant61) in LMS compared to in uterine smooth muscle, uterine fibroid cells. We also evaluated the synergic effect of SMO or GLI inhibitors with DNA Methyltransferases (DNMTs) inhibitor. Our *in vitro* study demonstrated that SMO and GLIs (1,2 and 3) expression was upregulated in LMS cells, with increased nuclear levels of GLI proteins compared to the uterine fibroid and uterine smooth muscle cells. Treatment with LDE225 (SMO inhibitor) and Gant61 (GLI inhibitor) resulted in a significant reduction in Glis protein levels in LMS cells. Additionally, the expression of DNMTs (1,3a and 3b) and GLI1 nuclear expression was significantly decreased after treatment with HH inhibitors in LMS cells. Our results showed that inhibition of SMO, GLI, and DNMTs is capable of suppressing LMS proliferation, migration, and invasion. Notably, the combination treatments exhibited a potentiated effect on LMS features *via* HH pathway deactivation [3].

To assess the effect of HH inhibitors on suppression of LMS *in vivo*, more recently, we evaluated the efficacy of HH inhibitors in a leiomyosarcoma xenograft model. Our results showed that although administration of SMO inhibitor (LDE225) in the animal model of LMS was inefficient to suppress the tumor growth, Gant61 as a GLI inhibitor exhibited a remarkable regression of the LMS growth with the significant decrease in the expression of Gli and GLI-target genes BMP4 and c-MYC.

## CONCLUSION

In conclusion, our studies demonstrate that HH signaling and epigenetic pathway are deregulated in leiomyosarcoma. Targeting the HH pathway and DNMTs may provide effective options for treating patients with leiomyosarcoma. Future studies are encouraged to use the LMS PDX model [4], and test the efficacy of HH inhibitors in combination with other drugs. The PDX model possesses several advantages, which allow for effective chronological tumor size monitoring, preserve tumor heterogeneity and lineage hierarchy, and potential applications in personalized medical treatments. In addition, conducting studies on the role and mechanisms of HH ligands (SHH, DHH, and IHH) as well as HH interaction with other vital pathways in LMS cells is needed. Furthermore, deeper mechanistic insights into LMS etiology and complexity genome widely will lead to elucidating and targeting resistance mechanisms and identifying more effective drugs for the treatment of patients with this aggressive cancer.
